# Mutations in the Methyltransferase Motifs of L Protein Attenuate Newcastle Disease Virus by Regulating Viral Translation and Cell-to-Cell Spread

**DOI:** 10.1128/Spectrum.01312-21

**Published:** 2021-09-29

**Authors:** Xiao Li, Lu Sun, Jing Zhao, Kaihang Tu, Jia Xue, Xin Guo, Guozhong Zhang

**Affiliations:** a Key Laboratory of Animal Epidemiology of the Ministry of Agriculture, College of Veterinary Medicine, China Agricultural University, Beijing, China; Wright State University

**Keywords:** Newcastle disease virus, negative-stranded RNA virus, large polymerase protein, K-D-K-E motif, viral translation, cell-to-cell spread

## Abstract

The large (L) polymerase proteins of most nonsegmented, negative-stranded (NNS) RNA viruses have conserved methyltransferase motifs, (G)-G-G-D and K-D-K-E, which are important for the stabilization and translation of mRNA. However, the function of the (G)-G-G-D and K-D-K-E motifs in the NNS RNA virus Newcastle disease virus (NDV) remains unclear. We observed G-G-D and K-D-K-E motifs in all NDV genotypes. By using the infection cloning system of NDV rSG10 strain, recombinant NDVs with a single amino acid mutated to alanine in one motif (G-G-D or K-D-K-E) were rescued. The intracerebral pathogenicity index and mean death time assay results revealed that the G-G-D motif and K-D-K-E motif attenuate the virulence of NDV to various degrees. The replication, transcription, and translation levels of the K-D-K-E motif-mutant strains were significantly higher than those of wild-type virus owing to their altered regulation of the affinity between nucleocapsid protein and eukaryotic translation initiation factor 4E. When the infection dose was changed from a multiplicity of infection (MOI) of 10 to an MOI of 0.01, the cell-to-cell spread abilities of G-G-D- and K-D-K-E-mutant strains were reduced, according to plaque assay and dynamic indirect immunofluorescence assay results. Finally, we found that NDV strains with G-G-D or K-D-K-E motif mutations had less pathogenicity in 3-week-old specific-pathogen-free chickens than wild-type NDV. Therefore, these methyltransferase motifs can affect virulence by regulating the translation and cell-to-cell spread abilities of NDV. This work provides a feasible approach for generating vaccine candidates for viruses with methyltransferase motifs.

**IMPORTANCE** Newcastle disease virus (NDV) is an important pathogen that is widespread globally. Research on its pathogenic mechanism is an important means of improving prevention and control efforts. Our study found that a deficiency in its methyltransferase motifs (G-G-D and K-D-K-E motifs) can attenuate NDV and revealed the molecular mechanism by which these motifs affect pathogenicity, which provides a new direction for the development of NDV vaccines. In addition to the (G)-G-G-D and K-D-K-E motifs of many nonsegmented, negative-stranded RNA viruses, similar motifs have been found in dengue virus, Zika virus, Japanese encephalitis virus (JEV), and severe acute respiratory syndrome coronavirus 2 (SARS-CoV-2). This suggests that such motifs may be present in more viruses. Our finding also provides a molecular basis for the discovery and functional study of (G)-G-G-D and K-D-K-E motifs of other viruses.

## INTRODUCTION

Only mature mRNA can be used as a template for protein synthesis. Maturation of mRNA is a rate-limiting step for the initiation of translation (1). This process involves capping and methylation of the 5′ end, the addition of a poly(A) tail at the 3′ end, and alternative splicing (2–4). Methylation of the mRNA cap structure is a common and important modification. In eukaryotes, mRNA modifications occur in the nucleus, and mature mRNA enters the cytoplasm as a template for protein synthesis. Viruses that replicate in the cytoplasm cannot utilize enzymes in the host nucleus. Virus capping and methylation often differ from their hosts, and viruses can have their own enzymes that catalyze mRNA methylation (5).

The large (L) polymerase protein of nonsegmented, negative-stranded (NNS) RNA viruses is a multifunctional protein that catalyzes genome replication, transcription, and mRNA modification (6). L protein has six conserved functional domains: CRI to CRVI (7). The specific functions of CRI, CRII, and CRIV have not yet been described. CRIII functions as an RNA-dependent RNA polymerase (8), CRV plays a role in adenylate polymerase and capping (9), and CR has methyltransferase activity (5, 10). Two methyltransferase motifs have been found in the L protein CR of NNS RNA viruses, the (G)-G-G-D motif and the K-D-K-E motif (10), which catalyze the methylation of G-N-7 and 2′-O sites of the mRNA cap structure (5). Methylated mRNA can bind to the eukaryotic translation initiation factor 4F (eIF4F), which is composed of eIF4E, eIF4G, and eIF4A (11–13). eIF4E can associate with the m^7^G cap structure, which is a key component in regulating translation initiation, eIF4G acts as scaffolding between eIF4E and eIF4A, and the function of eIF4A is to unhook the secondary structure at the 5′-untranslated region (UTR) of the mRNA, thus promoting scanning (14–16). The 2′-O methylation mainly mediates virus escape from innate immune recognition (17).

In recent years, many studies have been conducted on these two NNS RNA virus motifs. Mutation of these two motifs in vesicular stomatitis virus (VSV) resulted in changes to the virus plaque diameter and cap structure methylation levels (18). Mutations in the (G)-G-G-D motif of the *S*-adenosylmethionine (SAM)-binding sites of measles virus (MeV) and human metapneumovirus (hMPV) reduced virulence (19, 20). The function of these two motifs has also been determined in other viruses, such as Zika virus (ZIKV), dengue virus (DFV), and Japanese encephalitis virus (JEV) (21–23). The virulence of each of these viruses was decreased when the amino acid sites in their motifs were mutated to alanine. The (G)-G-G-D motif and K-D-K-E motif are targets for attenuating viruses. However, the precise mechanisms by which the (G)-G-G-D and K-D-K-E motifs regulate viral virulence and pathogenicity have not been well defined.

Newcastle disease virus (NDV), a member of the *Paramyxoviridae* family, is an NNS RNA virus (24). The disease caused by this pathogen, Newcastle disease (ND), is a highly contagious bird disease that is prevalent worldwide (25). Like other viruses that replicate in the host cytoplasm, NDV has motifs to complete the methylation of its own mRNA cap structures, including G-G-D and K-D-K-E motifs. However, the effect of these two motifs on the biological function of NDV remains unclear. NDV has only G-N-7 methylation modification and lacks 2′-O methylation modification (26). The aim of the present study was to investigate how the G-G-D and K-D-K-E motifs affect the virulence and pathogenicity of NDV. Our findings provide important insight into how methyltransferase motifs regulate the translation and cell-to-cell spread of NDV and could be targeted to attenuate NDV. This study demonstrates a previously uncharacterized role for methyltransferase motifs in the translation of NNS RNA viruses.

## RESULTS

### Rescue of the recombinant NDVs and analysis of their biological characteristics.

**FIG 1:**
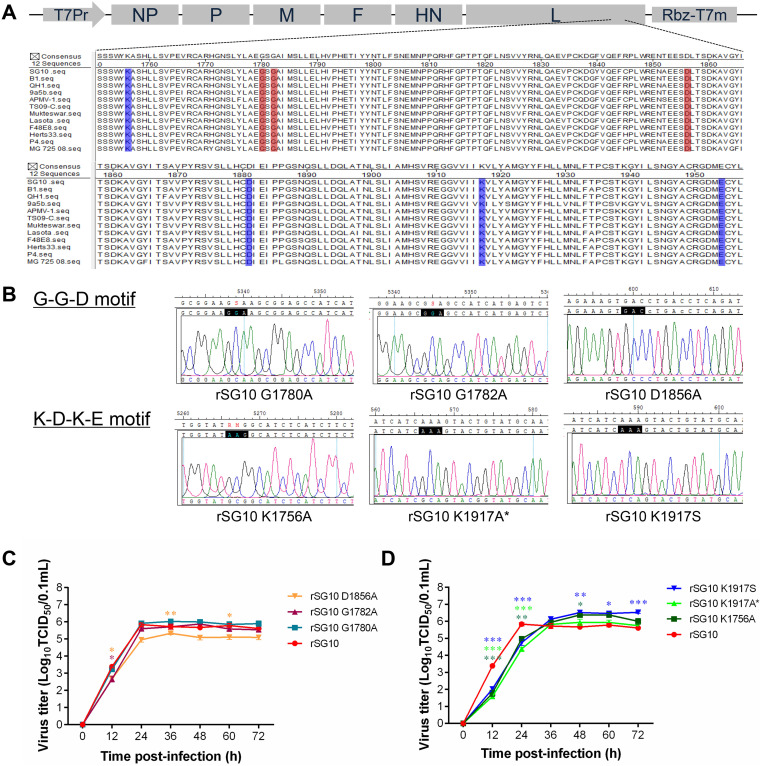
Rescue of rNDVs and detection of their biological characteristics. (A) Alignment of the NDV L protein amino acid sequences among different genotypes. The G-G-D motif is marked by a red rectangle, and the K-D-K-E motif is marked by a blue rectangle; F, fusion protein; HN, hemagglutinin-neuraminidase. (B) Sequencing of the mutant sites of the rNDV strains. The black rectangle represents the original codon, and the corresponding bottom shows the mutated codon. rSG10 K1917A* had one additional mutation site three bases away from the original mutation site: CTG-CGG. (C, D) Multicycle growth kinetics of rSG10 and the rNDVs with mutations in their K-D-K-E (C) or G-G-D (D) motif in DF1 cells infected at an MOI of 0.01. The supernatants of infected DF1 cells were collected at the indicated time points. Viral loads were quantified as the TCID_50_. *P* values were calculated with a two-way ANOVA; *n* = 3; *, *P* < 0.05; **, *P* < 0.01; ***, *P* < 0.001.

An amino acid sequence analysis of the predicted methyltransferase motifs in the L proteins of NDV strains with different genotypes revealed that the amino acids were the same as those in other viruses, except that the amino acid at site 1778 is an alanine (27) and that the amino acids at these seven sites (G-G-D and K-D-K-E) are conserved among various NDV genotypes (Fig. 1A).

To analyze the effect of the two predicted methyltransferase motifs on NDV, we attempted to rescue seven recombinant NDVs (rNDVs) with single-amino-acid mutations in each of the motif sites. However, after many attempts, only six rNDV strains could be successfully rescued: rSG10-G1780A, rSG10-G1782A, and rSG10-D1856A of the G-G-D motif and rSG10-K1756A, rSG10-K1917S, and rSG10-K1917A* of the K-D-K-E motif (Fig. 1B). The recombinant virus rSG10-K1917A was extremely unstable and mutated into two forms, rSG10-K1917A and rSG10-K1917A*, during passaging. The recombinant virus rSG10-K1917A* had an additional mutation at the L1919R site. Rapid reversion mutations occurred in the recombinant viruses rSG10-D1881A and rSG10-E1954A during passaging, and we were unable to recover these two viruses.

Multistep growth curves of the rNDV strains generated by infecting DF1 cells showed that the two groups of motif mutations had different growth trends. The three G-G-D motif rNDV strains showed growth trends similar to that of the wild-type rSG10, and there were no significant differences in the viral titers among these strains (Fig. 1C). The growth trends of the three K-D-K-E motif rNDV strains were similar to one another but different from that of rSG10. The titers of the K-D-K-E motif rNDVs were lower than that of rSG10 during the early phase and were higher than that of rSG10 after the peak at 48 h (Fig. 1D). These results indicate that the K-D-K-E motif may play an important role in the growth of NDV.

Intracerebral pathogenicity index (ICPI) and mean death time (MDT) assays were performed to determine the virulence of the rNDVs. The ICPI values of rSG10-G1780A, rSG10-G1782A, and rSG10-D1856A were all found to be approximately 1.6, which is approximately 0.2 less than that of the parental virus but still within the range associated with strong virulence. The ICPI values of rSG10-K1756A, rSG10-K1917A*, and rSG10-K1917S were all approximately 1.4, which indicates a moderate level of virulence. The MDTs of rSG10-G1780A, rSG10-G1782A, and rSG10-D1856A were similar to that of rSG10, whereas the MDTs of rSG10-K1756A, rSG10-K1917A*, and rSG10-K1917S were each greater than 90 h (Table 1). These results demonstrate that the virulence of rNDVs with a mutation in the G-G-D motif is slightly lower than that of rSG10, whereas the virulence of rNDVs with a mutation in the K-D-K-E motif is much lower.

**TABLE 1:** Virulence index of rSG10 wild-type and mutant viruses

Strains	rSG10	G-G-D motif			K-D-K-E motif		
		rSG10 G1780A	rSG10 G1782A	rSG10 D1856A	rSG10 K1756A	rSG10 K1917A*	rSG10 K1917S
ICPI score^*a*^	1.81	1.66	1.63	1.60	1.42	1.48	1.39
MDT (h)^*b*^	48	52	56	48	132	108	118

a Intracerebral pathogenicity index (ICPI): virulent strains, 1.50 to 2.00; moderately virulent strains, 0.70 to 1.50; avirulent strains, 0.00–0.70.

b Mean death time (MDT): virulent strains, <60 h; moderately virulent strains, 60 to 90 h; avirulent strains, >90 h.

### rSG10-K1756A showed opposite growth characteristics at different multiplicities of infection.

The growth characteristics and virulence of the three rNDV strains with mutations in the G-G-D motif were very similar to one another, as were those of the three rNDV strains with mutations in the K-D-K-E motif. For convenience of the study, we selected one mutant strain for each motif (rSG10-K1756A and rSG10-G1780A) for use in subsequent experiments.

**FIG 2:**
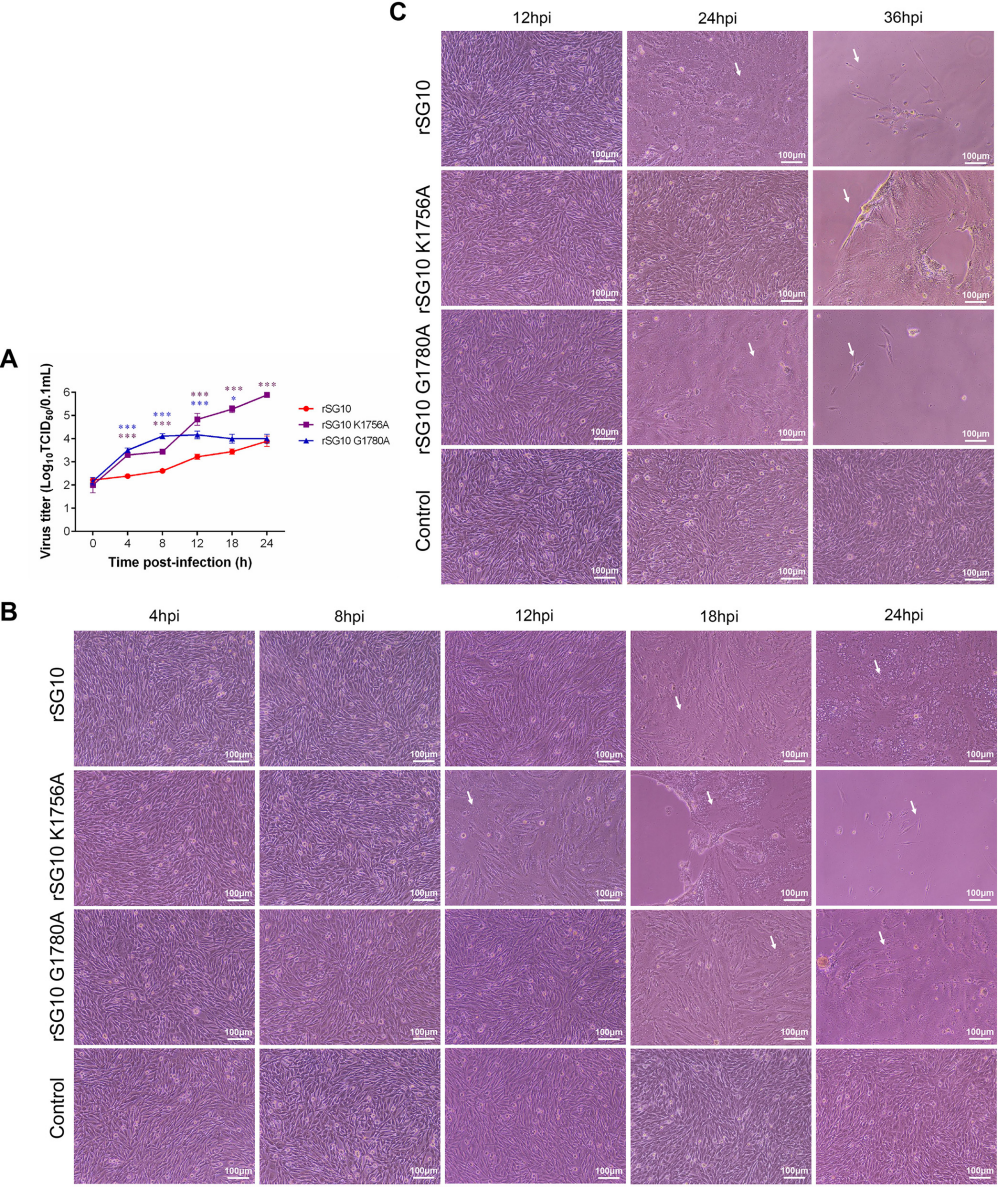
Reproduction of NDV strains in DF1 cells infected at different MOIs. (A) One-cycle growth kinetics of rSG10, rSG10-K1756A, and rSG10-G1780A in cells infected at an MOI of 10; *n* = 3; *, *P* < 0.05; ***, *P* < 0.001. (B, C) CPE caused by infection with the rNDV strains on DF1 cells at an MOI of 10 (B) or 0.01 (C). White arrows indicated CPE (where cells fall off or the cell structure disappears).

To test whether the above biological characteristics of rSG10-K1756A are dose dependent, we detected the one-step growth curves. The one-step growth curves of these rNDV strains were determined by infecting DF1 cells at a multiplicity of infection (MOI) of 10. Results showed that the viral titers of rSG10-K1756A were consistently higher than that of rSG10, whereas those of rSG10-G1780A were only higher than that of rSG10 during the early stage, and the titers of the two strains became similar after 12 h (Fig. 2A). This result indicates that the reproductive performance of rSG10-K1756A is clearly superior to that of rSG10 when infected at an MOI of 10, which was contrary to the data at an MOI of 0.01 in Fig. 1D.

**FIG 3:**
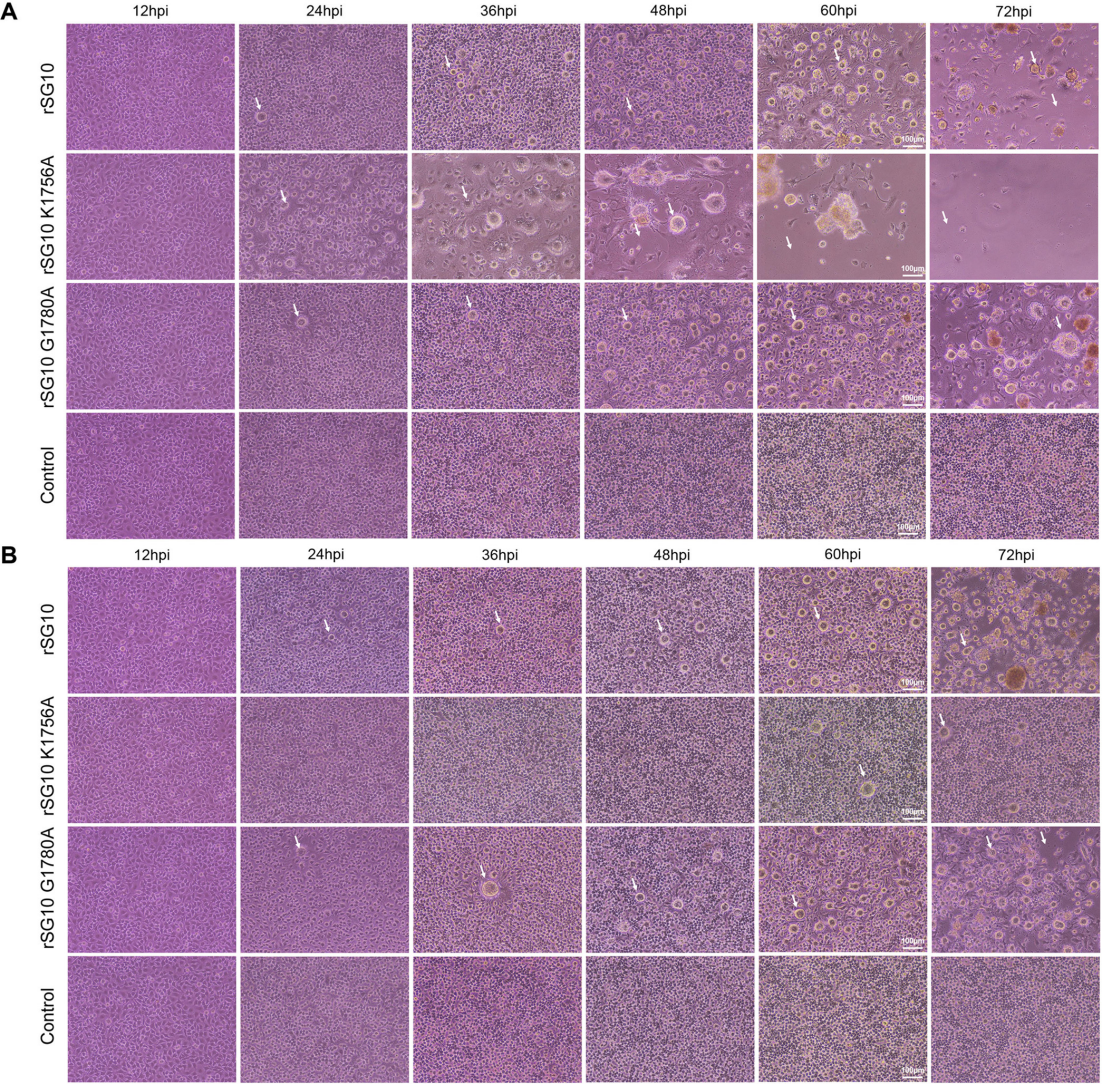
CPE caused by infection with the rNDV strains on BSR cells at an MOI of 1 (A) or 0.01 (B). White arrows indicated CPE (syncytium, where cells fall off, or where the cell structure disappears).

We then examined the cytopathic effect (CPE) produced by the rNDV strains at an MOI of 10 on DF1 cells at different postinfection time points. We observed that rSG10-K1756A had obvious CPE at 12 h, earlier than rSG10 or rSG10-G1780A. At 18 h and 24 h, the cells infected with rSG10 were blurred, and the cell structure disappeared; the cells infected by rSG10-G1780A were wrinkled with unclear boundaries, and some cells were shed to form gaps. These effects were much less extreme than those of rSG10-K1756A infection. Cells infected by rSG10-K1756A lost their original structure, and most of the cells died and fell off (Fig. 2B). But, CPE induced by rSG10-K1756A infection appeared later than that induced by rSG10 or rSG10-G1780A, and the associated lesions were mild and small when infected at an MOI of 0.01 (Fig. 2C). The trend of CPE produced by the rNDV strains in BSR cells was consistent with that in DF1 cells: when the infection dose was high, rSG10-K1756A was the earliest to cause CPE, while when the infection dose was low, the time of CPE appeared later in rSG10-K1756A-infected cells than in the other two infected groups (Fig. 3A and B). These results suggest that rSG10-K1756A could produce infection earlier when infected at an MOI of 10 and produce infection later when infected at an MOI of 0.01 in different cell lines.

### K1756A had opposite effects on replication, transcription, and translation of NDV at different MOIs.

**FIG 4:**
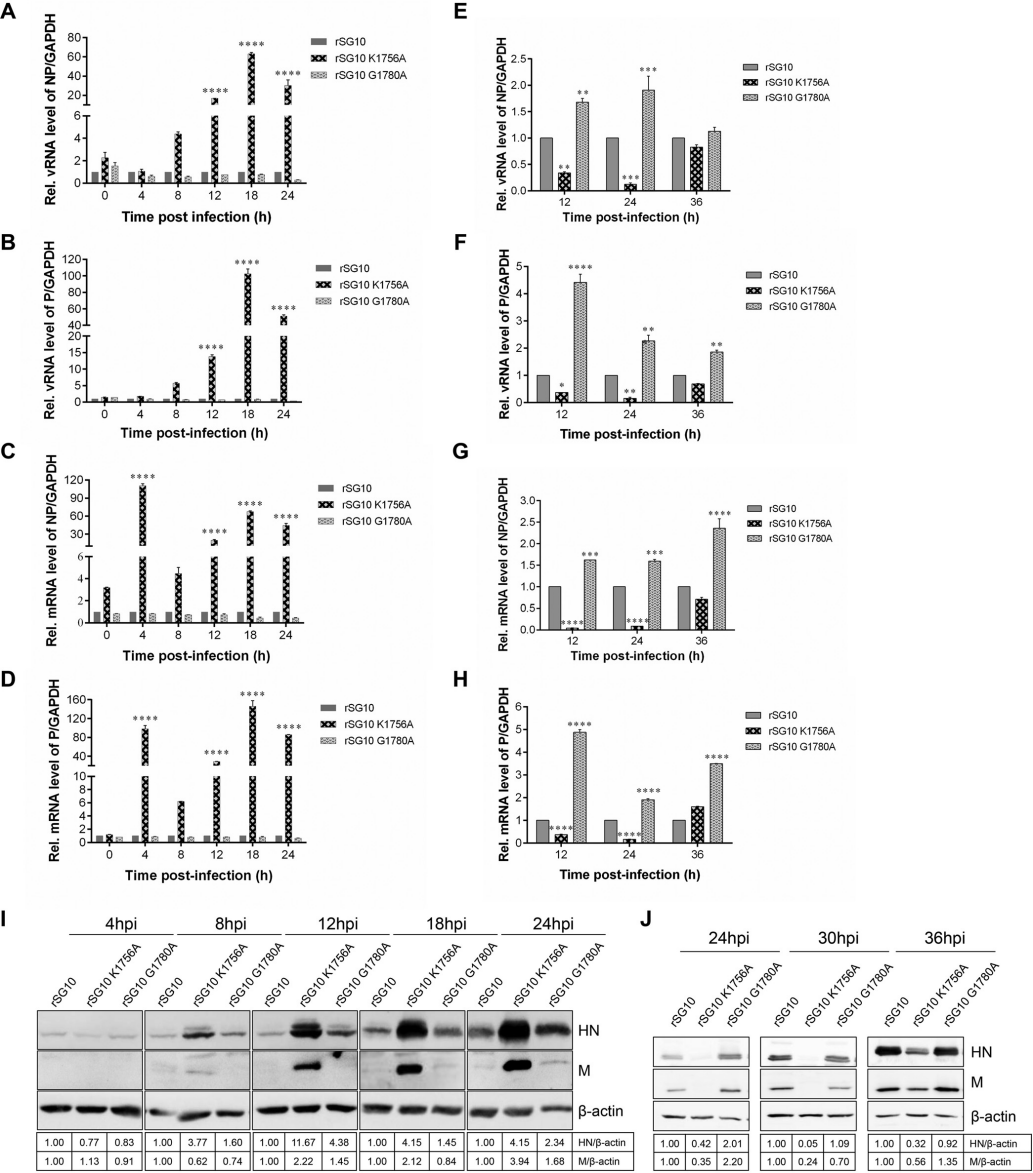
Replication, transcription, and translation levels of rNDVs at different MOIs. (A to D) Relative RNA expression levels of rNDV following DF1 cell infection at an MOI of 10. (E to H) Relative RNA expression levels of rNDV when an MOI of 0.01 was used for infection. The housekeeping gene used to normalize the results was *GAPDH*. The 2^−ΔΔ^*^CT^* value of rSG10 at each time point was defined as 1. The 2^−ΔΔ^*^CT^* values of rSG10-K1756A and rSG10-G1780A were compared with the 2^−ΔΔ^*^CT^* value of rSG10 at the corresponding time point. *P* values were calculated with a two-way ANOVA; *n* = 3; *, *P* < 0.05; **, *P* < 0.01; ***, *P* < 0.001; ****, *P* < 0.0001. (I, J) Western blotting was used to detect HN protein and M protein synthesis at different time points in cells infected with one of the rNDV strains at an MOI of 10 (I) or 0.01 (J). HN protein was detected by SG10 hyperimmune serum used at a dilution of 1:200. The relative grayscale value of the rSG10 protein band at each time point was defined as 1. The relative grayscale values of the rSG10-K1756A and rSG10-G1780A protein bands were compared with those of the rSG10 protein bands at the corresponding time point.

To further investigate the specific step that affects the reproductive performance of rSG10-K1756A, we measured the differences in replication, transcription, and translation between the rNDV strains and wild-type strain at different MOIs. First, we assessed the replication ability of the rNDV strains when infected with an MOI of 10 by detecting the level of viral RNA (vRNA). The results showed that from 12 h onward, the nucleocapsid protein (NP) and phosphoprotein (P) vRNA levels for rSG10-K1756A were significantly higher than those for rSG10, whereas there was no statistical difference between these levels for rSG10-G1780A and rSG10 (Fig. 4A and B). The mRNA results demonstrate that rSG10-K1756A had higher levels than rSG10, but rSG10-G1780A did not (Fig. 4C and D). These findings indicate that when infected with a high dose, rSG10-K1756A has superior replication and transcription abilities compared with rSG10, whereas rSG10-G1780A has replication and transcription abilities similar to those of rSG10. However, when an MOI of 0.01 was used for the infection dose, the vRNA and mRNA abundances of rSG10-K1756A were lower than those of rSG10 and rSG10-G1780A at 12 and 24 h postinfection (hpi) (Fig. 4E to H), which was opposite from the results when an MOI of 10 was used as the infection dose (Fig. 4A to D). At different postinfection time points, we collected cells to detect mRNA translation levels via Western blotting. Results showed that, from 8 hpi onward, the translation level of rSG10-K1756A was much higher than that of rSG10 whereas that of rSG10-G1780A was not when infected with an MOI of 10 (Fig. 4I). When the infection dose was an MOI of 0.01, the protein expression level of rSG10-K1756A was the lowest among the three tested strains (Fig. 4J). These results illustrated that the effect of the K1756A mutation on NDV replication, transcription, and translation was more substantial than that of the G1780A mutation. In summary, the replication, transcription, and translation advantages of rSG10-K1756A in DF1 cells disappeared when the infection dose was reduced from an MOI of 10 to 0.01.

### rSG10-K1756A activated cap-dependent translation more effectively at an MOI of 10.

**FIG 5:**
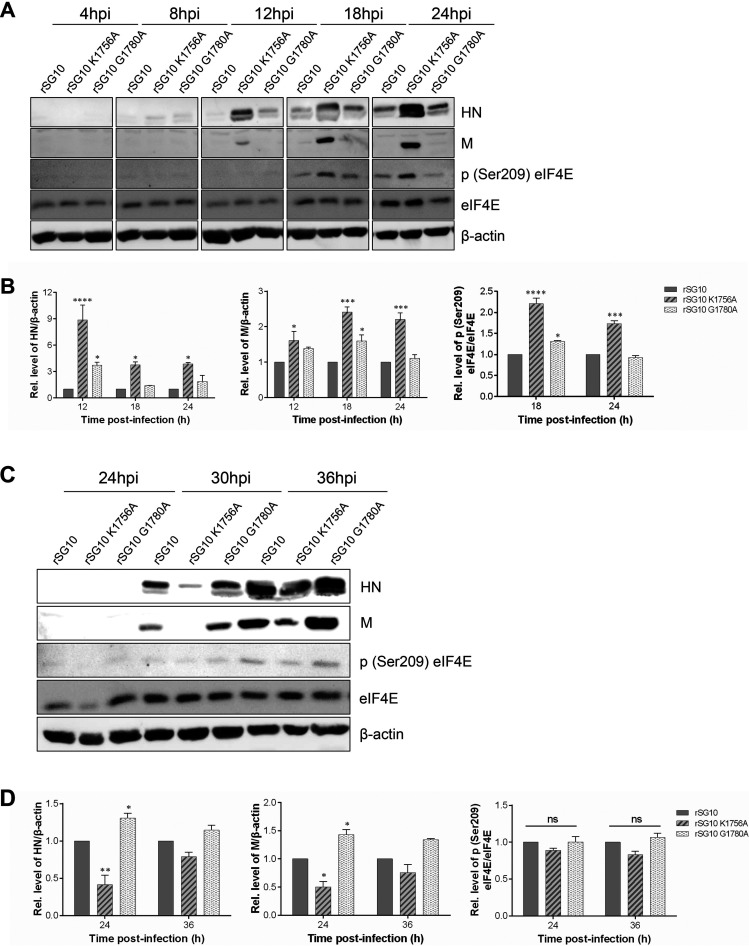
Detection of cap-dependent translation at different MOIs. (A) Differential analysis of phosphorylated (Ser209) eIF4E levels by Western blotting after infection at an MOI of 10. (B) Quantification of the phosphorylated eIF4E levels shown in A. (C) Differential analysis of phosphorylated (Ser209) eIF4E levels by Western blotting after infection at an MOI of 0.01. (D) Quantification of the phosphorylated eIF4E levels shown in C. The relative grayscale value of the rSG10 protein band at each time point was defined as 1. The relative grayscale values of the rSG10-K1756A and rSG10-G1780A protein bands were compared with that of the rSG10 protein band at the corresponding time point. *P* values were calculated with a two-way ANOVA; *n* = 3; ns, not significant; *, *P* < 0.05; **, *P* < 0.01; ***, *P* < 0.001; ****, *P* < 0.001.

As described above, we found that rSG10-K1756A had the opposite effect on replication, transcription, and translation compared with rSG10 when infected with different MOIs. The K-D-K-E motif is thought to influence the modification of cap structure methylation, which is important for the binding of eIF4E to mRNA; thus, we focused our research on the effect of the K-D-K-E motif on viral translation. To investigate the mechanism by which the K1756A mutation affects viral translation at different MOIs, we first assessed the difference in the activation of cap-dependent translation by detecting the level of eIF4E phosphorylation after infection with each of the rNDV strains because phosphorylation of eIF4E occurs after its binding to the cap structure to form eIF4F. The results showed that when the infection dose was an MOI of 10, rSG10-K1756A induced higher levels of eIF4E phosphorylation at 18 and 24 hpi than rSG10-G1780A and rSG10, accompanied by an increase in viral protein synthesis (Fig. 5A and B). These results demonstrated that when an MOI of 10 is used for infection, rSG10-K1756A can more effectively activate cap-dependent translation to promote viral protein synthesis. In light of the above results, we investigated whether the decrease in the amounts of vRNA, mRNA, and protein of rSG10-K1756A observed when an MOI of 0.01 was used for infection was caused by a decrease in cap-dependent translation. The results show that the phosphorylation level of eIF4E induced by rSG10-K1756A at different time points was not different from that induced by rSG10 or rSG10-G1780A at an MOI of 0.01 (Fig. 5C and D). These data suggest that the lower infection efficiency of rSG10-K1756A at an MOI of 0.01 is not because it influences translation efficiency. These data demonstrate that the higher level of replication, transcription, and translation of rSG10-K1756A after high-dose infection is due to its ability to activate cap-dependent translation effectively.

### rSG10-K1756A promoted cap-dependent translation via interaction of NP and eIF4E.

**FIG 6:**
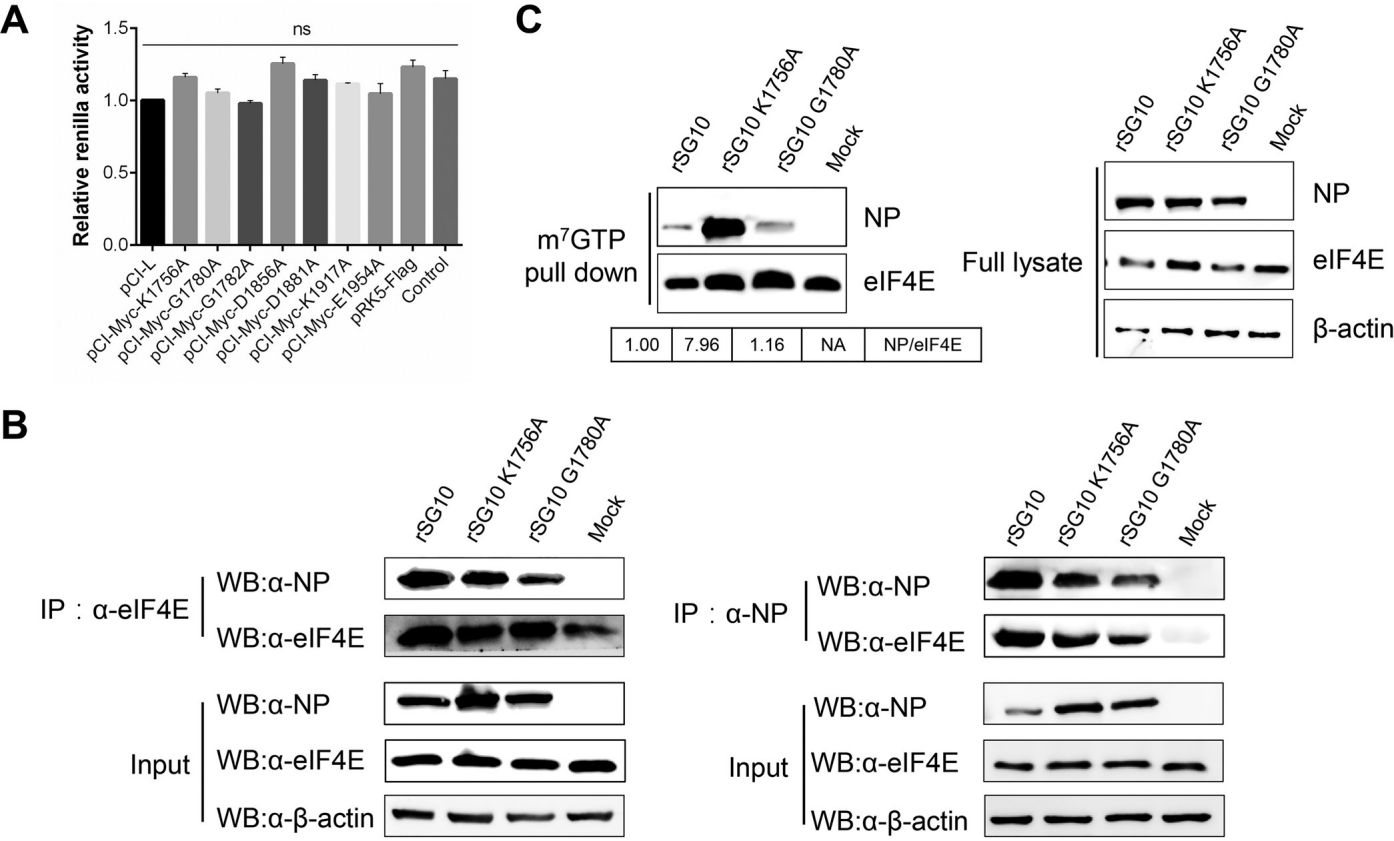
Detection of the interaction between NP and eIF4E after infection at an MOI of 10. (A) Translation inhibition assay. The renilla activity of rSG10 was defined as 1. *P* values were calculated with a one-way ANOVA; *n* = 3; ns, not significant. (B) Coimmunoprecipitation of NP and endogenous eIF4E during rSG10, rSG10-K1756A, or rSG10-G1780A infection in DF1 cells. Cells were used for immunoprecipitation with anti-NP antibodies or anti-eIF4E antibodies, and immunoblotting was performed with the indicated antibodies. (C) m^7^GTP pulldown was used to detect the affinity between NP and the cap structural analogue m^7^GTP during rSG10, rSG10-K1756A, or rSG10-G1780A infection. The protein band ratio of rSG10 NP/eIF4E was defined as 1.

We attempted to elucidate further the mechanism by which rSG10-K1756A promotes cap-dependent translation initiation after infection with high doses. We first performed translation inhibition assays as described in Materials and Methods and found no statistical differences among the expression of *Renilla* luciferase (RLuc) (Fig. 6A). These findings indicated that mutations in the amino acid sites of the methyltransferase motif do not affect viral translation directly but rather affect protein synthesis by regulating interactions between other proteins.

Previous reports have shown that the NDV NP protein can interact with eIF4E to regulate viral translation (28). From these results, we speculated that the K-D-K-E motif may regulate cap-dependent translation of the rNDV strains by affecting the affinity of NP and eIF4E. We performed coimmunoprecipitation to examine the interaction between NP and eIF4E. The results showed that NP could still interact with eIF4E after mutation of the K-D-K-E motif or G-G-D motif (Fig. 6B). We further quantitatively detected the amount of NP protein bound to the mRNA cap structure by m^7^GTP pulldown, and the results showed that the affinity between rSG10-K1756A NP protein and the cap structure was more robust than that observed in rSG10 and rSG10-G1780A (Fig. 6C). These results suggest that the K-D-K-E motif can affect the affinity between NP and the mRNA cap structure, which affects the affinity between NP and eIF4E, thereby regulating the activation of cap-dependent translation and upregulating the synthesis of viral proteins.

### Mutation of the G-G-D or K-D-K-E motif restrained cell-to-cell spread of NDV.

We found that the biological characteristics of rSG10-K1756A were significantly different between infection at an MOI of 10 and infection at an MOI of 0.01. We previously demonstrated that rSG10-K1756A propagated best at an MOI of 10 because the K1756A mutation can promote the binding of NP and eIF4E, thus activating higher levels of eIF4E phosphorylation, which enables the synthesis of viral proteins. However, there was no significant difference in eIF4E phosphorylation levels among the rNDV strains when an MOI of 0.01 was used for infection. The replication capacities of the viruses were detected following infection at an MOI of 10, and the outcome of infection at an MOI of 0.01 was determined by examining both viral replication and cell-to-cell spread. Because the replication ability of rSG10-K1756A was enhanced during infection at an MOI of 10, we speculated that the reduced reproductive characteristic of rSG10-K1756A during infection at an MOI of 0.01 might be the result of a decline in cell-to-cell spread ability.

**FIG 7:**
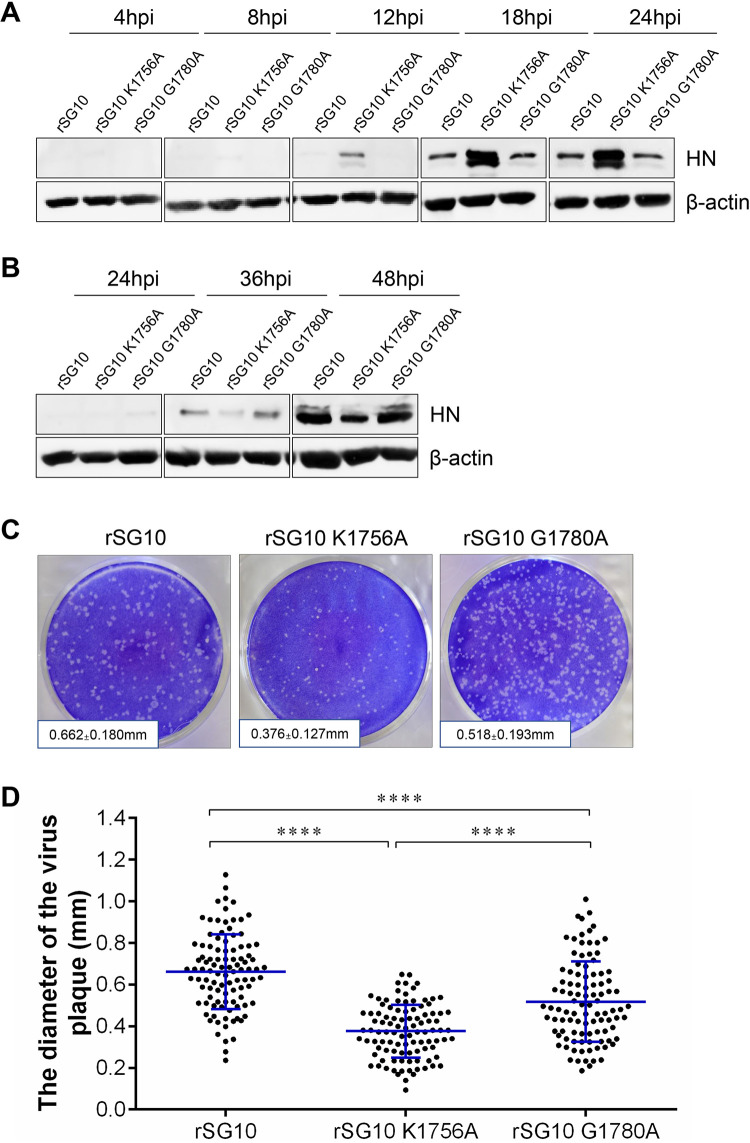
Cell-to-cell spread ability of NDV strains. (A, B) Expression levels of HN protein during infection of BSR T7/5 cells with different NDV strains at an MOI of 10 (A) or 0.01 (B). (C) Diameter of plaques formed in BSR T7/5 cells by different NDV strains. The plaque diameters are shown as the means ± SD. (D) The diameters of 100 plaques per group were analyzed statistically. The black lines represent the mean ± SD. *P* values were calculated with a one-way ANOVA; ****, *P* < 0.0001.

We confirmed our hypothesis by performing a plaque assay in BSR T7/5 cells. First, we verified that protein expression of the rNDVs was not cell type dependent at MOIs of 10 and 0.01. The results showed that protein expression by the rNDVs was consistent between DF1 cells and BSR T7/5 cells; the highest level of protein expression for rSG10-K1756A was observed at the high-infection dose, and the lowest level of protein expression was observed at the low-infection dose (Fig. 7A and B). Next, we performed a plaque assay in BSR T7/5 cells, the results of which showed that the plaque diameter was 0.662 ± 0.180 mm following infection with rSG10, 0.376 ± 0.127 mm following infection with rSG10-K1756A, and 0.518 ± 0.193 mm following infection with rSG10-G1780A (Fig. 7C). The plaque diameter for rSG10 was the largest and was significantly larger than those for rSG10-G1780A and rSG10-K1756A. The plaque diameter for rSG10-G1780A was smaller than that for rSG10 but significantly larger than that for rSG10-K1756A (Fig. 7D). Thus, the cell-to-cell spread ability of rSG10-G1780A is inferior to that of rSG10 but superior to that of rSG10-K1756A, and the cell-to-cell spread ability of rSG10-K1756A is the weakest among the three strains. This result is consistent with our hypothesis.

**FIG 8:**
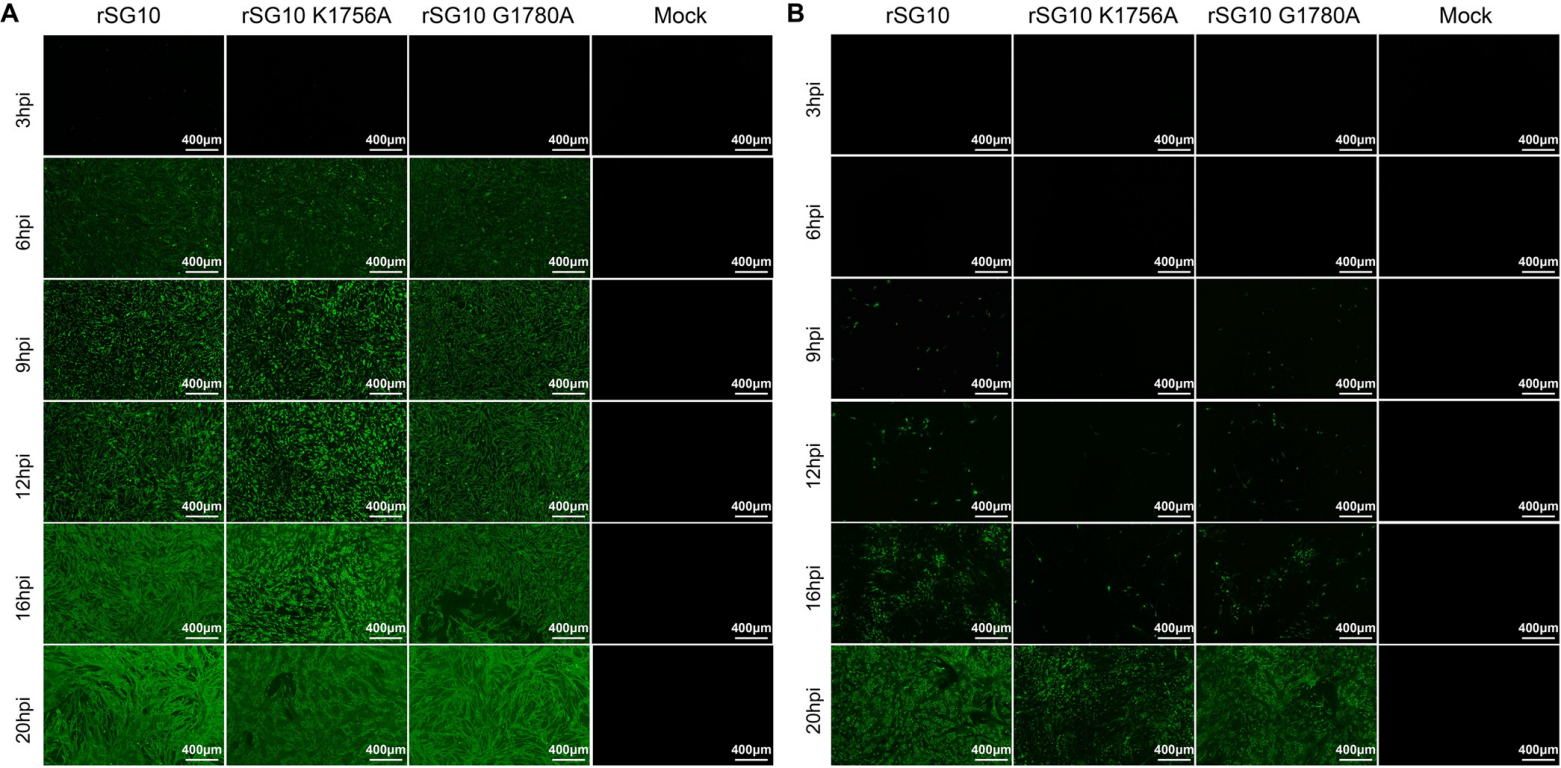
Dynamic distribution of viruses during infection at different MOIs. Cells were infected with NDV strains at an MOI of 10 (A) or 0.01 (B). Anti-NP antibodies were used to detect the NP protein of the viruses.

We further detected dynamic distribution of the viruses by performing indirect immunofluorescence assays. When an MOI of 10 was used for infection, the three rNDVs could infect nearly all the cells simultaneously, fluorescence could be observed at 6 hpi, and the fluorescence intensity of the three viruses gradually increased until 20 hpi (Fig. 8A). When an MOI of 0.01 was used for infection, green fluorescence could be detected in the rSG10 infection group at 9 hpi, after which the fluorescence level gradually increased until 20 hpi, and fluorescence could be seen in almost all cells. For the rSG10-G1780A infection group, fluorescence was also detected from 9 hpi, and its levels gradually increased over time, but they were lower than those in the rSG10 infection group. For the rSG10-K1756A infection group, weak fluorescence could be observed at 12 hpi, but the level of fluorescence was much lower than that in the rSG10 and rSG10-G1780A infection groups; at 20 hpi, the fluorescence range of rSG10-K1756A still did not cover all cells (Fig. 8B). These results indicate that, compared with rSG10, both rSG10-G1780A and rSG10-K1756A had inferior cell-to-cell spread abilities, especially rSG10-K1756A. In summary, the G-G-D and K-D-K-E motifs play an important role in NDV cell-to-cell spread.

**FIG 9:**
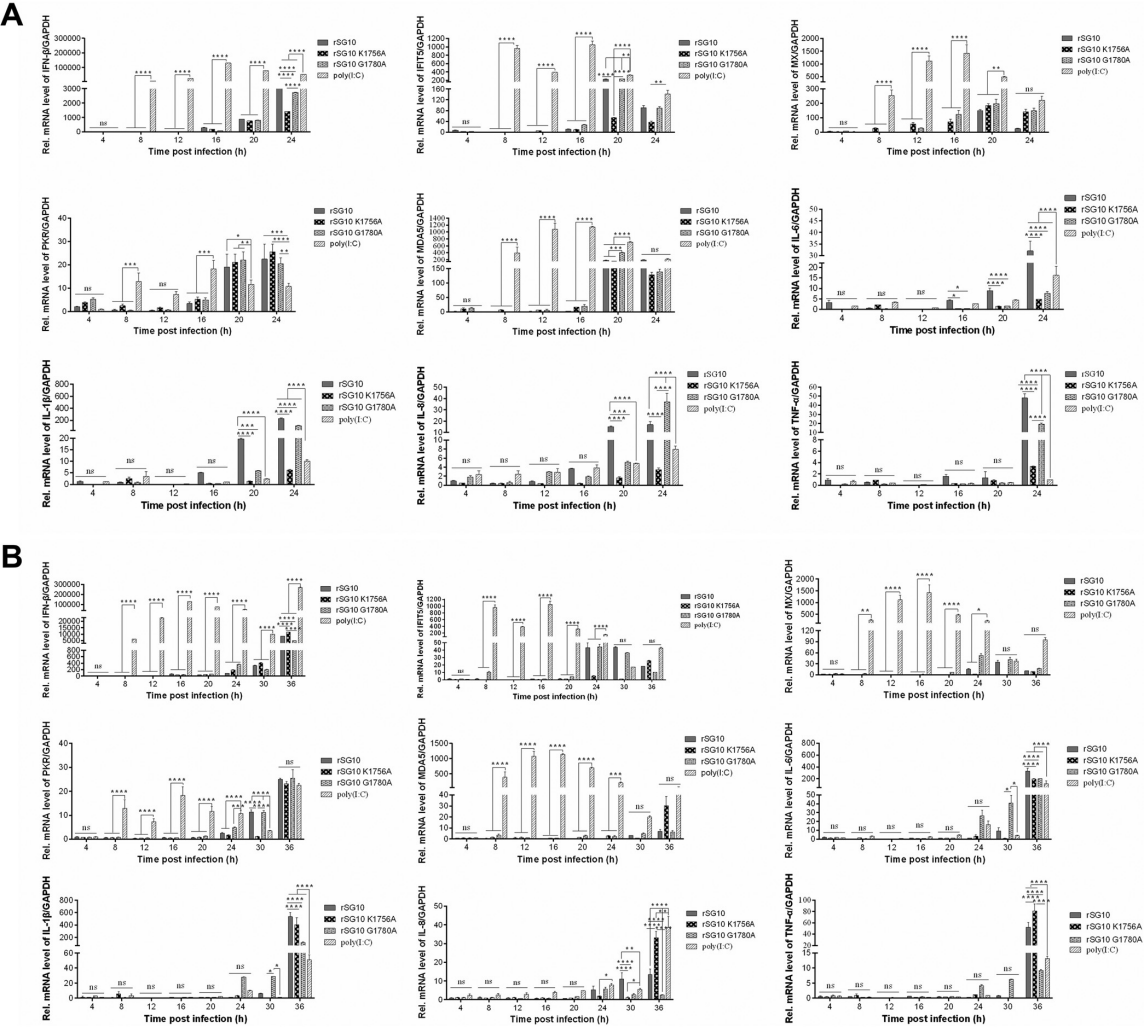
Antiviral response induced by viruses at different MOIs. DF1 cells were infected with rNDV strains at an MOI of 10 (A) or 0.01 (B). Poly(I·C) (1,000 ng) was added as a positive control. Cells were harvested at each time point, and mRNA levels of IFN-β, interferon induced protein with tetratricopeptide repeats 5 (IFIT5), myxovirus resistance protein (MX), protein kinase R (PKR), melanoma differentiation-associated protein 5 (MDA5), interleukin-6 (IL-6), IL-1β, IL-8, and tumor necrosis factor-α (TNF-α) were detected by qRT-PCR. The housekeeping gene used to normalize the results was *GAPDH*. The 2^−ΔΔ^*^CT^* value of the control at each time point was defined as 1. The 2^−ΔΔ^*^CT^* values of rSG10, rSG10-K1756A, rSG10-G1780A, and poly(I·C) were compared with the 2^−ΔΔ^*^CT^* value of the control at the corresponding time point. *P* values were calculated with a two-way ANOVA; *n* = 3; ns, not significant; *, *P* < 0.05; **, *P* < 0.01; ***, *P* < 0.001; ****, *P* < 0.0001.

Viral infection induces a robust antiviral response in host cells. After different doses of infection, the status of intracellular antiviral response was different. We measured levels of interferon (IFN), interferon-stimulating genes (ISGs), and cytokines (CKs) to assess the impact of antiviral responses on the cell-to-cell spread of the virus (Fig. 9A and B). The results showed that there was no strong antiviral response in the early stage after virus infection except for the positive-control poly(I·C) stimulation group. Differences in levels of IFN, ISGs, and CKs induced after infection of the three strains were detected only in the late stage of infection. Differences in the levels of replication, transcription, and translation of the virus appeared earlier than the differences in antiviral responses. These results demonstrate that the antiviral response is not responsible for the discrepancy in viral translation and spread ability after high- and low-dose infection.

### Mutations in the G-G-D or K-D-K-E motif attenuated NDV *in vivo*.

**FIG 10:**
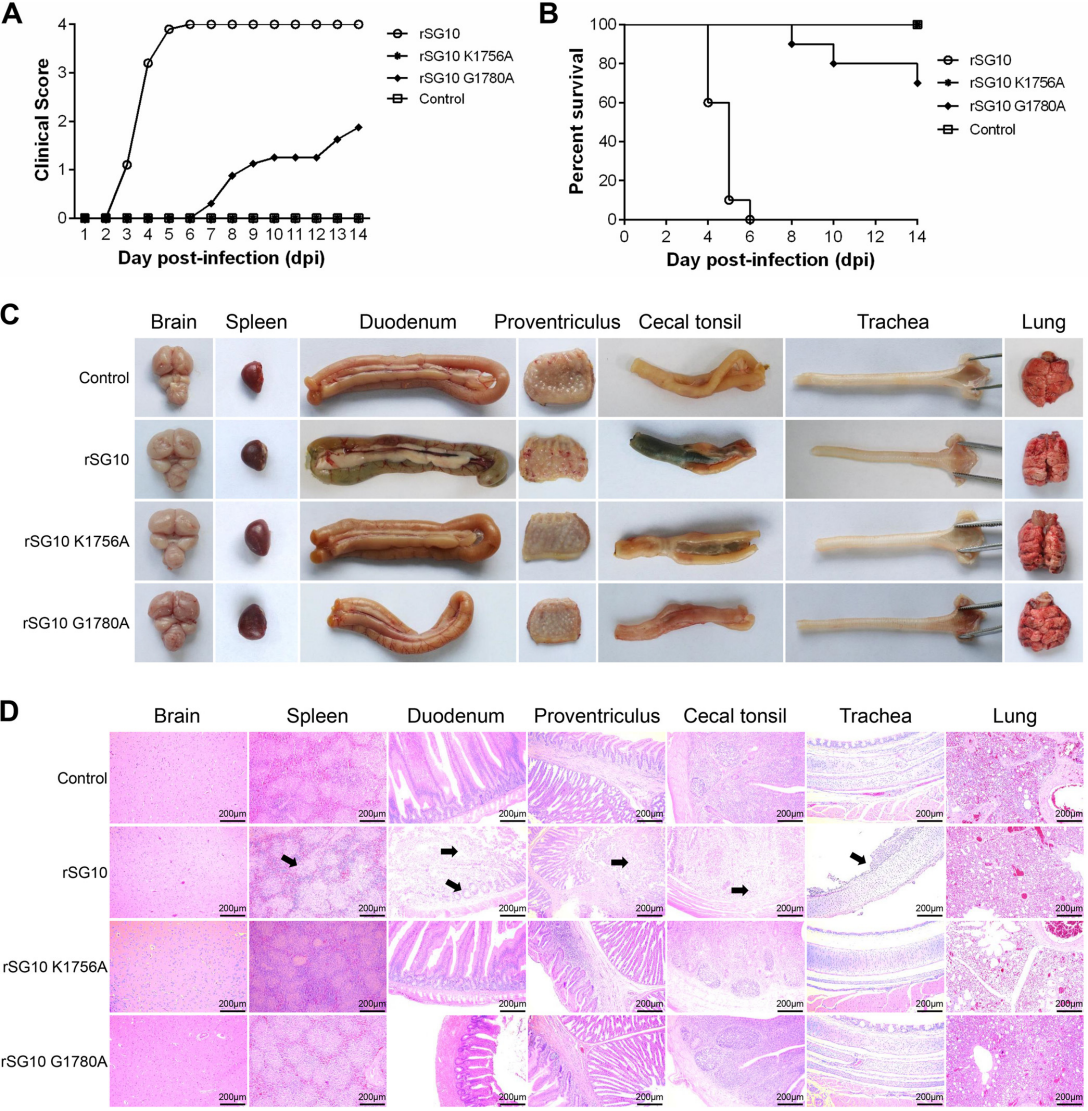
Pathogenicity of rNDV strains in 3-week-old SPF chickens. (A) Clinical signs in the NDV-infected chickens were scored daily (0, healthy; 1, sick; 2, wing drop, paralysis, torticollis, or lack of coordination; 3, prostration; 4, death). (B) Survival of 3-week-old SPF chickens, calculated from 10 birds per group. (C) Organ lesions of chickens inoculated with rSG10, rSG10-K1756A, or rSG10-G1780A. (D) Tissue histopathology of NDV-inoculated 3-week-old chickens. Birds were euthanized at 5 dpi, and their tissues were fixed in formalin, sectioned, and stained with hematoxylin and eosin. Pathological changes are marked with a black arrow.

The pathogenic and replication abilities of the rNDVs were investigated *in vivo*. Three-week-old, specific-pathogen-free (SPF) chickens were infected via the oculonasal route with wild-type or mutated NDV strains at a 50% egg infective dose (EID_50_) of 10^4.0^ per bird. Chickens infected with rSG10 began showing clinical signs of ND at 3 days postinfection (dpi), began to die at 4 dpi, and reached 100% mortality at 6 dpi. In the rSG10-G1780A infection group, chickens began showing weakness and depressed behavior at 7 dpi. During the observation period, three chickens died, one each at 8, 10, and 14 dpi, yielding a final mortality rate of 30%. No clinical signs of ND or death were observed in the rSG10-K1756A infection or control groups (Fig. 10A and B). As expected, the rSG10-infected chickens presented typical symptoms of ND, such as bleeding of the proventriculus and duodenum. No obvious organ lesions were observed in the other groups (Fig. 10C).

Three chickens from each group were euthanized at 5 dpi for histopathological analysis (Fig. 10D). Infection with the parental strain rSG10 caused severe histological changes, including lymphocyte necrosis and reduced lymphocyte numbers in the spleen, severe mucosal epithelial shedding and necrosis in the proventriculus, villus shedding and decreased numbers of intestinal glands in the duodenum, a large area of severe intestinal mucosal necrosis in the cecum, and reduced lymphoid cells and severe mucosal shedding in the trachea. No pathological changes were observed in the rSG10-K1756A or rSG10-G1780A infection groups or in the control group.

**FIG 11:**
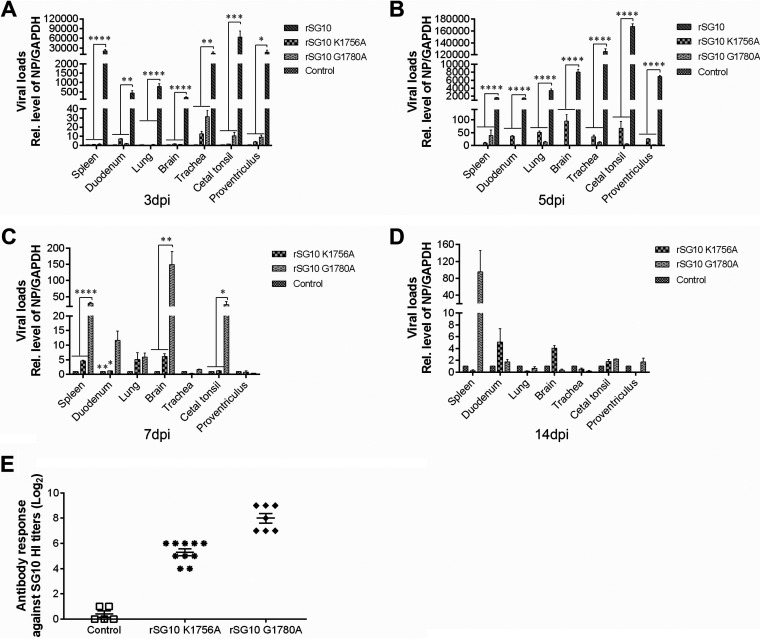
Replication ability of and specific antibody levels induced by the rNDV strains *in vivo*. (A to D) Three NDV-inoculated birds of each group were euthanized at 3 dpi (A), 5 dpi (B), 7 dpi (C), or 14 dpi (D). The indicated tissues were collected, and their viral loads were determined by qRT-PCR. The 2^−ΔΔ^*^CT^* value of the control group for each tissue was defined as 1. The 2^−ΔΔ^*^CT^* values of the rSG10-K1756A, rSG10-G1780A, and rSG10 groups were compared with the 2^−ΔΔ^*^CT^* value of the control group. *P* values were calculated with a two-way ANOVA; *n* = 3; *, *P* < 0.05; **, *P* < 0.01; ***, *P* < 0.001; ****, *P* < 0.0001. (E) Serum antibody titers of chickens surviving through 14 dpi were detected by hemagglutination inhibition (HI).

We then used reverse transcription-quantitative PCR (qRT-PCR) to determine the viral loads in chicken organs. At 1 dpi, no virus was detected in any organs from the chickens in any group. At 3 dpi, high viral loads were detected in all organs of the rSG10 infection group, whereas only a small amount of virus was detected in a few organs (trachea, proventriculus, duodenum, and cecal tonsils) of individual chickens in the rSG10-K1756A and rSG10-G1780A infection groups (Fig. 11A). At 5 dpi, virus was detected in all organs of chickens in the rSG10 infection group, whereas only a small amount of virus was detected in some organs of the rSG10-K1756A and rSG10-G1780A infection groups (Fig. 11B). At 7 dpi, all chickens in the rSG10 infection group died. Virus was detected in the spleen, brain, and cecal tonsils of chickens in the rSG10-G1780A infection group, and these viral loads were statistically different from those in the rSG10-K1756A infection group (Fig. 11C). At the end of the 14-day observation period, chickens in the rSG10-G1780A and rSG10-K1756A infection groups had low viral loads, with only a small viral load in the spleen of one chicken in the rSG10-G1780A infection group (Fig. 11D).

To detect virus shedding, cloacal swabs were collected from chickens at various postinfection time points. From 3 dpi onward, all chickens in the rSG10 infection group expelled the virus to the environment through the cloaca, and the shedding rate was 100%. In the rSG10-G1780A infection group, viral shedding was detected in only two chickens at 7 dpi. No viral shedding was detected in the rSG10-K1756A infection and control groups (Table 2). At the end of the observation period, we collected blood from live chickens in each group to verify their antibody titers. The titers of anti-NDV antibody in the rSG10-K1756A infection group were between 4 and 6, whereas the titers of the rSG10-G1780 infection group were between 7 and 9, and the average titer was 3 units higher than that of the rSG10-K1756A infection group (Fig. 11E). These results demonstrate that mutations in the G-G-D motif and K-D-K-E motif attenuated NDV *in vivo*, which indicates that these motifs are very important for the pathogenicity of NDV.

**TABLE 2:** Shedding rates of chickens after NDV challenge

Group	Shedding rate (positive no./total no.)			
	3 dpi	5 dpi	7 dpi	14 dpi
rSG10	6/6	1/1	NA^*a*^	NA^*a*^
rSG10 K1756A	0/10	0/10	0/10	0/7
rSG10 G1780A	0/10	0/10	2/10	0/7
Control	0/10	0/10	0/10	0/10

a NA, not available.

## DISCUSSION

The L protein (G)-G-G-D and K-D-K-E motifs are conserved among NNS RNA viruses and are closely related to methylation of the mRNA cap structure (18, 27). Previous studies on these motifs have examined various NNS RNA viruses, but none have focused on NDV (10, 19, 27, 28). Here, we rescued rNDVs with single-amino-acid mutations in their G-G-D and K-D-K-E motifs. Although it was straightforward to obtain three rNDV strains with single-amino-acid mutations in the G-G-D motif, we faced challenges in our attempt to generate rNDV strains with single-amino-acid mutations in the K-D-K-E motif. After multiple rounds of plaque purification, the rSG10-K1756A strain was obtained. Our attempts to produce rSG10-K1917A yielded two different mutants, rSG10-K1917A* and rSG10-K1917S. We were unable to produce rSG10-D1881A and rSG10-E1954A despite many attempts, and we speculate that these two amino acid mutations were lethal for NDV. Similar issues have been reported in studies conducted on other viruses; for example, when attempting to rescue JEV K61A-D146A-K182A-E218A motif-mutant strains, D146A and K182A were found to be fatal mutations (23). Furthermore, for MeV, G1790A- and G1698A-mutant viruses were not able to be rescued (20). Together, these findings suggest that different amino acids are of different importance to different viruses. For NDV, the rescue of the K-D-K-E motif-mutant strains either failed or presented some difficulty. Therefore, we speculated that the K-D-K-E motif may be more important to NDV than the G-G-D motif and that the loss of its function may have a greater impact on NDV. The MDT and ICPI results confirm this hypothesis.

Previous studies demonstrated that the (G)-G-G-D and K-D-K-E motifs also have different effects on the growth of VSV, JEV, and avian metapneumovirus (aMPV). The growth peak of some recombinant strains on cells was delayed, and the CPE occurrence time was later than that of the wild-type virus when an MOI of 0.01 or a 50% tissue culture infective dose (TCID_50_) of 20 was used for infection (18, 23, 27). Additionally, no difference in protein expression was detected in the recombinant strains of JEV with a K-D-K-E motif mutation (23). However, our results demonstrate that a mutation in the K-D-K-E motif of NDV had a significant inhibitory effect on the growth of NDV when an MOI of 10 was used for infection, which is different from the effect of a mutation in the K-D-K-E motif of other viruses. Although previous studies performed infection using a lower viral dose, we used an MOI of 10 for infection to observe one cycle of infection. The difference in infection dose may be the reason why our results are inconsistent with those of other studies. Notably, when an MOI of 0.01 was used for infection, the rNDV strains with a K-D-K-E motif mutation showed a growth delay phenomenon.

The methyltransferase motif can modify the mRNA cap structure via methylation (5, 22, 23), and modification of the cap structure is closely related to its combination with eIF4E. NDV replicates in the cytoplasm, hijacking the translation apparatus of the host cell to synthesize viral proteins (29). Other studies have focused on either the effect of methyltransferase motifs on the level of methylation of the viral mRNA cap structure and pathogenicity or on the mechanism by which the interaction between eIF4F and viral protein affects viral translation (30, 31). Here, we explored both questions in an effort to reveal the effect of NDV methyltransferase motifs on viral translation. We hypothesized that the NDV methyltransferase motifs may directly affect the translation of viral proteins by altering the affinity between the cap structure and eIF4E and may indirectly affect the levels of replication and transcription via changing the level of translation. Phosphorylation of eIF4E occurs following its recruitment to the cap structure for eIF4F assembly (32). Because the Ser209 phosphorylation site of eIF4E is very close to its cap-binding pocket, the phosphorylation of eIF4E may enhance its ability to bind to the cap structure (33). Thus, the detection of post-rNDV infection changes in eIF4E phosphorylation level can be used to assess the level of activation of cap-dependent translation by the rNDV strains. When we measured the phosphorylated (Ser209) eIF4E level induced by infection with each of the rNDV strains, we found that rSG10-K1756A was able to activate higher levels of cap-dependent translation.

NDV infection activates the phosphatidylinositol 3-kinase (PI3K)/Akt/mechanistic target of rapamycin (mTOR) and p38 mitogen-activated protein kinase (MAPK)/MAPK-interacting protein kinase 1 (MnK1) pathways to benefit viral mRNA translation via interaction between NP and eIF4E (29). The NP-eIF4E interaction is critical for mRNA translation. Our results suggest that a deficiency in K-D-K-E motif function promotes NP-eIF4E binding, thereby altering the phosphorylation level of eIF4E and promoting the translation of viral mRNA. Our findings provide a new research direction for the effect of the K-D-K-E motif on the translation ability of viruses.

Our study results also provide insight into the ability of rNDV to spread from cell to cell. Unexpectedly, when the infection dose was changed from an MOI of 10 to 0.01, rSG10-K1756A exhibited very weak proliferation that was independent of the eIF4E phosphorylation level. This finding prompted us to investigate the mechanisms responsible for differences in the biological characteristics of rSG10-K1756A at different infection doses. According to the Poisson distribution, at an MOI of 10, there are theoretically enough virions to infect every cell, whereas at an MOI of 0.01, the virions could infect only a subset of cells. In this case, to infect the rest of the cells, the first batch of viruses would need to complete a replication cycle and assemble into complete progeny viruses before starting the next round of infection. The results generated from infections produced at an MOI of 0.01 are regulated by both replication and cell-to-cell spread capacity. The effect of the K-D-K-E and G-G-D motifs on the intercellular spread of NDV was confirmed by our plaque assay and dynamic indirect immunofluorescence assay results. After mutation of the VSV methyltransferase motifs, the diameter of plaques produced by some recombinant strains was significantly reduced (18). Additionally, a (G)-G-G-D motif mutation of aMPV also results in a decrease in plaque diameter (27). Furthermore, the loss of methyltransferase function led to smaller plaque diameter for both MeV and JEV (20, 23), which is consistent with our findings. Thus, the methyltransferase motifs (G)-G-G-D and K-D-K-E have an important role in promoting the cell-to-cell spread of many viruses.

We tried to uncover how the methyltransferase motifs regulate virus spread from cell to cell and have ruled out the antiviral response as a significant factor. Many viruses can be spread from cell to cell by tunneling nanotubes (TNTs) (34, 35). We preliminarily found that rNDVs can also be spread through TNTs. The efficiency of TNT utilization of the recombinant strains was different, suggesting that TNTs might be a factor affecting the cell-to-cell spread ability of NDV. More experiments are needed to verify our hypothesis and further reveal the detailed molecular mechanisms.

Previous work has shown that mutations in the (G)-G-G-D and K-D-K-E motifs can lead to attenuated pathogenicity (20, 27). Here, a pathogenicity assay conducted in 3-week-old SPF chickens showed that rSG10-K1756A had a lower level of pathogenicity than rSG10-G1780A, which had a lower level of pathogenicity than rSG10; these findings are consistent with our ICPI and MDT data. During the 14-day observation period, the chickens in the rSG10-K1756A infection group did not show any clinical symptoms, and anti-NDV antibodies could be detected in these chickens at the end of this period, which indicates that rSG10-K1756A caused infection but not disease in these chickens. In contrast, some chickens in the rSG10-G1780A infection group exhibited clinical symptoms of ND and died after 7 dpi, which indicates that the pathogenesis of the rSG10-G1780A strain was delayed. Together with the plaque assay results, these findings demonstrate that the cell-to-cell spread ability of rSG10-G1870A was inferior to that of rSG10. The weakened cell-to-cell spread ability of rSG10-G1780A likely led to the slow diffusion of rSG10-G1780A in chickens and the consequent delay in pathogenesis. Regarding the pathogenicity assay, inoculating the chickens with an EID_50_ of 10^4.0^ introduced a relatively small number of virus particles for 3-week-old chickens, and the immune response in the body is stronger than that in isolated cells. After the viral particles entered through the mucosal pathway, the generation of infection in the body required multiple rounds of reproduction and cell-to-cell spread; thus, the process was more similar to the situation in which cells were infected with NDV at an MOI of 0.01. This may also explain why the K-D-K-E motif-mutant rNDV strains reproduced best at an MOI of 10 but had lower virulence and pathogenicity.

Because the G-G-D and K-D-K-E motifs are located on the L protein, which is not a target of neutralizing antibodies, the immunogenicity of the rescued recombinant viruses should theoretically not be greatly affected by mutations to these regions. Infection with rSG10-K1756A or rSG10-G1780A could induce the production of NDV-specific antibodies in chickens. Our rNDV strains achieved high viral titers on DF1 cells, BSR T7/5 cells, and Vero cells (data not shown); therefore, they could be suitable for mass production if they prove useful for vaccination in future studies. Additionally, our study demonstrated that the loss of function in the cap structure of NDV mRNA caused by mutation to the methyltransferase motif had an impact on the viral virulence; given that most viral mRNAs have a methylated modified cap structure (28), the development of broad-spectrum antiviral drugs that target the formation and modification of the viral cap structure might be useful clinically.

Motifs similar to the NDV methyltransferase motifs are present in the nonstructural protein 5 (NS5) protein of many members of the flavivirus family, which are more harmful to human health (22, 34). In addition, the nonstructural protein 14 (nsp14) proteins of severe acute respiratory syndrome coronavirus (SARS-CoV), middle east respiratory syndrome coronavirus (MERS-CoV), and SARS-CoV-2 have methyltransferase activity (35–37), and Sudan ebolavirus (SUDV) and human immunodeficiency virus (HIV) also have methyltransferase motifs (38–40). There may be many viral methyltransferase motifs that have not yet been discovered. Our study could provide a scientific basis for revealing the function of methyltransferase motifs in other viruses, which has the potential to aid in drug design and vaccine development.

In conclusion, our findings show that the two methyltransferase motifs G-G-D and K-D-K-E regulate the efficiency of translation and cell-to-cell spread ability of NDV, thus affecting viral virulence and pathogenicity. This study also revealed the molecular mechanism by which the G-G-D and K-D-K-E motifs affect the pathogenicity of NDV and provided evidence for a mechanism by which other (G)-G-G-D and K-D-K-E motifs may affect the protein synthesis and pathogenicity of NNS RNA viruses. Our work serves as a reference for the study of additional attenuated viral strains generated through a loss of methyltransferase motif function. Further research on the mechanisms of viral pathogenesis is needed to provide new directions for vaccine development and disease prevention and control.

## MATERIALS AND METHODS

### Cells and viruses.

DF-1 cells (a chicken embryo fibroblast cell line), Vero cells (an African green monkey kidney cell line), and BSR T7/5 cells (a baby hamster kidney cell line stably expressing T7 RNA polymerase) were cultured in Dulbecco’s modified Eagle’s medium (DMEM; Gibco, Grand Island, NY, USA) with 10% fetal bovine serum (FBS; Gibco) and were maintained in DMEM with 2% FBS at 37°C in a 5% CO_2_ incubator (Thermo Forma, Marietta, OH, USA). The rNDV strain rSG10 was generated in our laboratory (41) and propagated in 9- to 11-day-old SPF embryonated eggs by allantoic cavity inoculation.

### Antibodies.

Mouse monoclonal antibody against matrix (M) protein and mouse polyclonal antibody against nucleocapsid (NP) protein were prepared in our laboratory. Anti-Myc, anti-β-actin, anti-eIF4E, and other primary antibodies were from Cell Signaling Technology (Beverly, MA, USA). The secondary antibodies against mouse, rabbit, or chicken used for indirect immunofluorescence assays and Western blotting were purchased from Bioss Biotechnology (Beijing, China).

### Animals and ethics statement.

SPF embryonated eggs (9 to 11 days old) and SPF chickens (1 day or 3 weeks old) were purchased from Beijing Boehringer Ingelheim Vital Biotechnology Co., Ltd. (Beijing, China). The animal experimental protocol was approved by the Beijing Administration Committee of Laboratory Animals under the auspices of the Beijing Association for Science and Technology (approval ID SYXK [Jing] 2018-0038) and Ethical Censor Committee at China Agricultural University (CAU approval no. 2019056).

### Plasmid construction and virus rescue.

A plasmid encoding the full-length antigenomic cDNA of rSG10 and three helper plasmids encoding the NP, P, and L proteins (pCI-NP, pCI-P, and pCI-L, respectively) were all constructed previously (41, 42). pCI-L was used as a PCR template to amplify cDNA encoding full-length wild-type L protein with a Myc tag at its C terminus; the resulting product was cloned into pCI-neo to generate pCI-Myc-L. cDNA encoding the different G-G-D and K-D-K-E motifs with various mutations were cloned into pCI-neo to generate the following plasmids: pCI-Myc-K1756A, pCI-Myc-G1780A, pCI-Myc-G1782A, pCI-Myc-D1856A, pCI-Myc-D1881A, pCI-Myc-K1917A, and pCI-Myc-E1954A. To construct full-length infectious clones containing single-site mutations of the G-G-D and K-D-K-E motif amino acid sites, the sequences containing mutant sites between MIuI and NotI were obtained by overlap PCR and then replaced on a pOK-SG10 vector. Virus rescue was performed as previously described (41).

### Cell transfection and infection.

Cells grown to approximately 70% to 80% confluence were transfected with the indicated plasmids by using Lipofectamine 2000 (Thermo Fisher Scientific, Waltham, MA, USA) and collected at the indicated time points. For infection, cells were infected with NDV at an MOI of 0.01 or 10. After virus absorption at 37°C for 1 h, the culture medium were removed, and the cells were washed three times with phosphate-buffered saline (PBS). The cells were then cultured in maintenance medium at 37°C. Viral titers on the cells were determined as the median tissue culture infective dose (TCID_50_), as described previously (42).

### Growth kinetics.

The growth kinetics of the NDV strains were determined using one-cycle or multiple-cycle growth kinetics in DF-1 cells. Cells in triplicate wells of 24-well culture plates were infected with viruses at an MOI of 0.01 or 10. The culture supernatants were collected at indicated time points. Viral titers in the collected supernatants were measured with limiting dilution using the endpoint method and are shown as the TCID_50_.

### Intracerebral pathogenicity index.

Ten 1-day-old SPF chicks were inoculated via the intracerebral route with 50 μl of a 10× dilution of fresh allantoic fluid infected with one of the rNDVs. The birds were monitored for clinical symptoms and mortality every 24 h for 8 days. The following scoring criteria were used: normal, 0; sick, 1; dead, 2. The ICPI value is the mean of the scores per bird over the 8-day observation period.

### Mean death time.

Infected allantoic fluid was made through a series of 10-fold dilutions with sterile PBS. Five 9-day-old SPF eggs were inoculated with 100 μl of each dilution via the allantoic cavity and then incubated at 37°C for 6 days. The time at which the mortality of each embryo was first observed was recorded. The highest dilution yielding 100% mortality was considered to be the minimum lethal dose. The MDT was determined as the mean time (h) required for the minimum lethal dose of the virus to kill all the inoculated SPF chicken eggs.

### qRT-PCR.

At indicated time points, total RNA was extracted and reverse transcribed to cDNA; qRT-PCR was performed on this cDNA with Premix *Ex Taq* reagents (TaKaRa, Dalian, China). Primer sequences were derived from previous reports (43). The comparative threshold cycle (ΔΔ*C_T_*) method was used to calculate the relative abundance of mRNA and vRNA. All experiments were performed in triplicate. The expression levels of the target genes encoding the NP and P proteins were detected and normalized to those of the gene encoding GAPDH.

### Indirect immunofluorescence assay.

Cells were harvested at indicated time points, washed three times with PBS, and then incubated with primary antibody at 4°C overnight. The cells were subsequently washed three times with PBS (5 min/wash) at room temperature and then incubated with fluorescein isothiocyanate (FITC)-conjugated secondary antibodies (Bioss Biotechnology; diluted 1:200) at 37°C for 1 h. The nuclei were stained with 4′,6-diamidino-2-phenylindole (DAPI; Sigma-Aldrich, St. Louis, MO, USA). The cells were washed five times with PBS (5 min/wash) and then observed and photographed on a Nikon Ti2-E fluorescence microscope (Nikon, Tokyo, Japan).

### Translation inhibition assay.

Translation inhibition assays were performed in BSR T7/5 cells that had been cotransfected with the expression vectors for RLuc and one of the following plasmids: pCI-L, pCI-Myc-K1756A, pCI-Myc-G1780A, pCI-Myc-G1782A, pCI-Myc-D1856A, pCI-Myc-D1881A, pCI-Myc-K1917A, and pCI-Myc-E1954A. At 30 hpi, the cells were harvested and washed three times with PBS. After being suspended in 100 μl of lysis buffer (Promega, Madison, WI, USA), the cells were vigorously mixed for 15 min, and 20 μl of lysate from each well was incubated with 30 μl of Stop&Glo Reagent from a dual-luciferase assay kit (Promega) to determine the renilla activity level.

### Western blotting.

DF1 cells were infected with rSG10, rSG10-K1756A, or rSG10-G1780A, and then harvested at indicated time points. Protein samples were obtained from these cells by using the ProteinExt mammalian total protein extraction kit (TransGen, Beijing, China). The protein samples were separated with 10% SDS-PAGE and transferred to a polyvinylidene difluoride (PVDF) membrane (Amersham Biosciences, Freiburg, Germany). The membranes were blocked with 5% (wt/vol) skim milk and 0.1% Tween 20 in Tris-buffered saline (TBST) for 2 h at 37°C and then incubated at 4°C overnight with primary antibody. After being washed three times with TBST, the membranes were incubated for 1 h at room temperature with horseradish peroxidase (HRP)-conjugated secondary antibodies (Bioss Biotechnology; 1:10,000 dilution). Protein bands were visualized using enhanced chemiluminescence (ECL) Western blotting detection reagents (CWBIO, Beijing, China). Protein abundance was quantified by using Image J-win64.

### Coimmunoprecipitation.

DF1 cells were separately infected with each of the different NDV strains. The cells were harvested at indicated time points, lysed with Pierce IP lysis buffer (Thermo) containing 1 mM phenylmethylsulfonyl fluoride (PMSF) (Solarbio, Beijing, China), and then centrifuged at 12,000 × *g* for 10 min. The resulting supernatant was incubated with indicated primary antibodies overnight at 4°C, and the complexes were pulled down by incubation with GammaBind G Sepharose (GE Healthcare, Waukesha, WI, USA) for 8 h. The coprecipitation samples and whole-cell lysates were analyzed by Western blotting.

### γ-Aminophenyl-m^7^GTP (C_10_-spacer)-agarose (m^7^GTP) pulldown assay.

DF1 cells were separately infected with each of the different NDV strains at an MOI of 10 and harvested at 20 hpi. The cells were lysed with Pierce IP lysis buffer (Thermo) containing 1 mM PMSF (Solarbio) and then centrifuged at 12,000 × *g* for 10 min. After determining the protein concentration in the resulting supernatant, 800 mg of protein was incubated overnight at 4°C with 40 μl of γ-aminophenyl-m^7^GTP (C_10_-spacer)-agarose (Jena Bioscience GmbH, Jena, Germany). After this incubation, the beads were washed three times with precooled PBS. The washed beads were boiled in 2× SDS protein loading buffer, and samples were analyzed by Western blotting.

### Pathogenicity in 3-week-old chickens.

The pathogenicities of rSG10, rSG10-K1756A, and rSG10-G1780A were determined in chickens. One hundred 3-week-old SPF chickens were assigned randomly into four groups of 25 birds each (15 for sampling, and 10 for clinical observation). The chickens in each group were inoculated with rSG10, rSG10-K1756A, or rSG10-G1780A at an EID_50_ of 10^4^ per bird via the oculonasal route. The negative-control group was mock infected with PBS. Over the 14-day observation period, the birds were observed daily and scored for the following clinical signs: healthy, 0; sick, 1; wing drop, paralysis, torticollis, or lack of coordination, 2; prostration, 3; dead, 4 (44). At 1, 3, 5, and 7 dpi, three birds from each group were euthanized, and spleen, proventriculus, duodenum, cecum tonsil, lung, brain, and trachea samples were collected for viral load detection via qRT-PCR.

### Statistical analyses.

All data were analyzed with GraphPad Prism software version 5.0 (GraphPad Software Inc., San Diego, CA, USA). All values shown in the manuscript express the mean ± standard deviation (SD) of three independent experiments. One-way and two-way analysis of variance (ANOVA) tests were used to evaluate the significance of differences.
